# No association between vitamin D deficiency at admission and outcome in a medical ICU

**DOI:** 10.1186/cc10760

**Published:** 2012-03-20

**Authors:** M Claus, L Schmitz, A Roman, E Stevens, P Dechamps

**Affiliations:** 1 Hôpital Saint Pierre, Brussels, Belgium

## Introduction

Vitamin D deficiency is associated with chronic illness and an excess in morbidity and mortality in the general population. Studies have found an even higher prevalence in ICU patients. The aim of our study was to evaluate the relationship between 25-OH vitamin D deficiency at admission and the outcome in a medical ICU.

## Methods

A prospective observational study in a 10-bed medical ICU at an inner-city hospital in Brussels. Patients with an expected stay in ICU >48 hours were included.

## Results

Vitamin D deficiency was defined as a serum 25-OH vitamin D concentration <20 ng/ml. The study was conducted between February and August 2011. A total of 105 patients were included. Dosages were performed on day 3 (2, 4) (median, interquartiles). The number of patients with 25-OH vitamin D <10 ng/ml, between 10 and 20 ng/ml, between 20 and 30 ng/ml and >30 ng/ml was respectively 56, 26, 14 and 9. No differences were seen between deficient and nondeficient patients if we compare SAPS III (58 ± 13 vs. 60 ± 15), predicted mortality (34 ± 21% vs. 40 ± 25%), intra-ICU mortality (8.5 vs. 8.7%), intrahospital mortality (19.5 vs. 21.7%), mean length of stay in the ICU (10 days ± 8), and median SOFA score during the first 5 days (5, 4, 4, 3, 3 vs. 4, 4, 3, 3, 4). A higher (but nonsignificant) prevalence of sepsis was found at admission in deficient patients (42/82 patients vs. 8/23 patients). Eleven deficient patients were treated with oral vitamin D (25,000 units/day) for 5 days. After treatment, 25-OH vitamin D was above 20 ng/ml in seven patients (31 ± 14 ng/ml). If we adjust groups for vitamin D post treatment, no differences were found if we compare deficient versus nondeficient patients for intra-ICU mortality (9.3% vs. 6.6%) and intra-hospital mortality (14.6% vs. 23.3%).

## Conclusion

Our study confirmed the high prevalence of vitamin D deficiency in ICU patients but not the association with an excess of mortality.
